# Sebaceous Adenoma: A Dermoscopic Case Perspective

**DOI:** 10.7759/cureus.49126

**Published:** 2023-11-20

**Authors:** Jesus Ivan Martinez-Ortega, Felipe de Jesus Perez Hernandez, Itzel Anayn Flores-Reyes, Ricardo Quiñones-Venega, Ilse Fernández-Reyna, Jorge Arturo Valdivieso-Jimenez

**Affiliations:** 1 Department of Dermatology, Dermatological Institute of Jalisco "Dr. José Barba Rubio", Zapopan, MEX; 2 Department of Internal Medicine, Regional High-Specialty Hospital of the Yucatan Peninsula, Mérida, MEX; 3 Department of Internal Medicine, Hospital General "Dr. Agustin O'Horan", Mérida, MEX

**Keywords:** skin tumor, rainbow like image, dermoscopy, muir-torre syndrome, sebaceous adenoma

## Abstract

This report focuses on sebaceous skin tumors, specifically sebaceous adenoma, sebaceoma, and sebaceous carcinoma, along with their association with Muir-Torre syndrome (MTS). A clinical case of a 25-year-old male with a suspected sebaceous neoplasm based on dermoscopy appearance is presented. The histopathological examination confirmed the diagnosis and surgical management resulted in successful treatment. The report highlights the importance of considering differential diagnoses and utilizing dermoscopy for accurate evaluation of these rare skin tumors.

## Introduction

Sebaceous skin tumors can be classified into three types: sebaceous adenoma, sebaceoma, and sebaceous carcinoma. These tumors are uncommon [1]. In this case study, we present a clinical case with suspected characteristics based on dermoscopy appearance. Although these tumors are generally benign, it is crucial to carefully consider the differential histopathological diagnosis and their potential association with Muir-Torre syndrome (MTS). Moreover, the significance of dermoscopy in evaluating this neoplasm should not be underestimated.

## Case presentation

**Figure 1:**
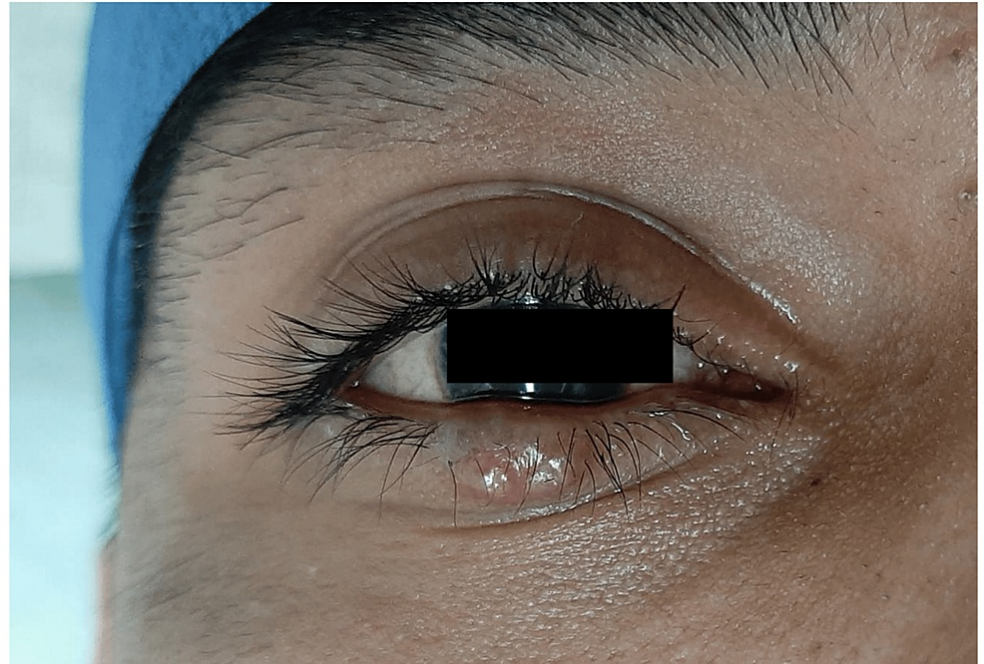
Clinical lesion on the right lower eyelid

A 25-year-old male presented with a "lump" in the lower right eyelid that had been present for approximately four years. The patient reported discomfort when closing the eye. There was no relevant medical history or previous treatments. On physical examination, a dome-shaped neoformation measuring 1.3x0.9x0.3 cm was observed. It exhibited light yellow and skin-colored areas, a pearly appearance, and telangiectasias on the surface. The lesion had well-defined, raised edges on the lower right eyelid (Figure 1).

**Figure 2:**
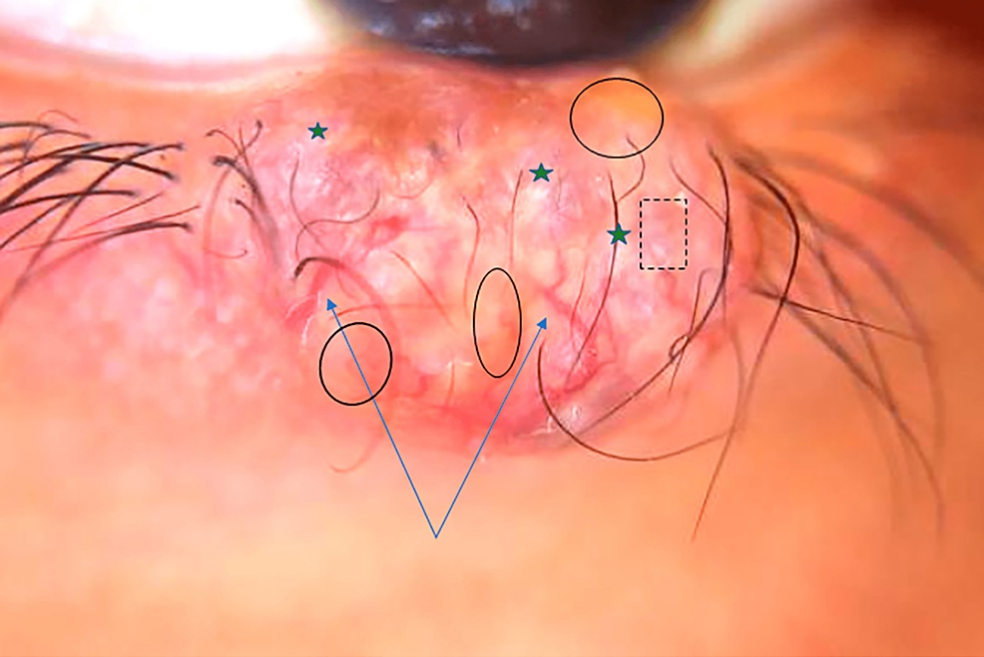
Dermoscopy of the lesion The lesion exhibits non-melanocytic features, including arborizing vessels (blue arrows), whitish structures resembling cotton flakes (dark blue stars), a yellow-orange background typical of sebaceous components (black circles), and subtle hints of a rainbow-like image (dotted rectangle).

Dermoscopy revealed a non-melanocytic lesion with arborizing vessels, whitish structures resembling cotton flakes, a yellow-orange background typical of sebaceous components, and hints of a rainbow-like image. (Figure 2).

**Figure 3:**
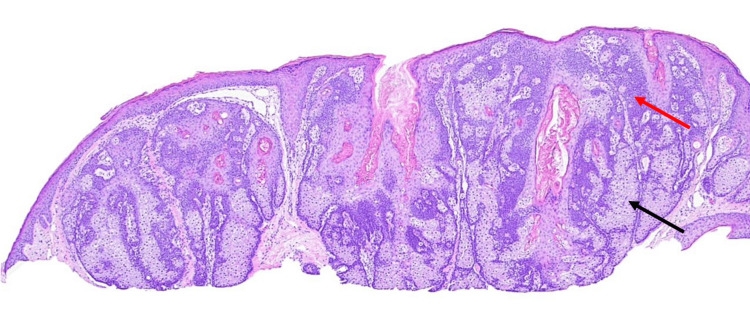
Hematoxylin and eosin stain The dermis shows multiple well-circumscribed lobules comprising an admixture of basaloid cells (indicated by red arrows) and mature sebocytes (indicated by black arrows).

Histopathological examination using hematoxylin and eosin staining showed acanthosis on the epidermis, hyperpigmentation within the basal layer, and a proliferation of epithelial cells with sebaceous differentiation, confirming the diagnosis of sebaceous adenoma (Figure 3).

**Figure 4:**
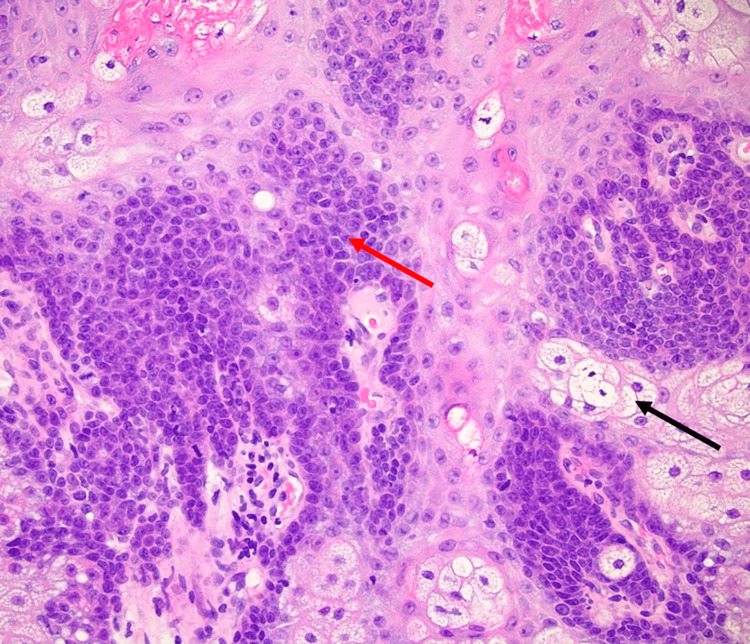
Hematoxylin and eosin stain at 40x Magnified view at 40x showing the basaloid cells (red arrow) and mature sebocytes (black arrow).

These cells were grouped and exhibited an irregular shape and size, resembling a grape (Figure 4) [2].

The patient underwent surgical management, which involved a wedge resection of the eyelid with a 3 mm safety margin of healthy skin. The defect was repaired using an advancement flap. During the three-month post-surgery follow-up, there was no recurrence.

## Discussion

Sebaceous tumors present challenges in comprehending their pathogenesis and genetic profile, as these aspects remain limited. However, a widely accepted theory suggests that alterations in the Wnt/beta-catenin signaling pathway play a crucial role in their development. Studies on transgenic mice, expressing a defective beta-catenin binding site in the lymphoid enhancer-binding factor (LEF-1) transcription factor, have demonstrated the spontaneous development of sebaceous skin tumors. Mutations in the LEF-1gene have been observed in sebaceomas and sebaceous adenomas, while sebaceous carcinomas may exhibit complete silencing of the LEF-1 gene [1,2].

In individuals with MTS and sebaceous gland neoplasias, germline mutations in DNA mismatch repair genes, particularly MSH2, have been reported. However, there seems to be no direct correlation between the presence of LEF-1 mutations and the loss of MSH2 expression, suggesting that they are not directly linked [3].

Sebaceous adenoma is the most common sebaceous tumor associated with MTS [4]. These rare benign neoplasms originate from the epithelial tissue and are characterized by well-defined irregular lobules within the papillary dermis. Clinically, they usually emerge in the head and neck region, appearing as small erythematous or yellowish lesions, sometimes with crusting or an umbilicated appearance. In the eyelids, they can involve the glands of Zeiss or the meibomian glands. Sebaceous adenomas typically manifest around the age of 60 and usually measure approximately 5 mm in their greatest dimension [5]. Dermatoscopic features of sebaceous adenoma are still limited in the literature. However, one study involving 18 cases suggested two possible dermatoscopic patterns. The first pattern is characterized by tumors with a central crater, exhibiting elongated radial telangiectasias (crown vessels) surrounding an opaque structureless ovoid white-yellow center, which can sometimes be covered by blood crusts. The second pattern is observed in tumors without a central crater, revealing branching but unfocused arborizing vessels over a white-to-yellow background, with a few loosely arranged yellow comedo-like globules. These yellow ovoid structures correspond histopathologically to dermal conglomerations of enlarged sebaceous glands, a feature shared by all sebaceous lesions [6]. In addition, these comedo-like globules may vary in form; in our case, they resemble more "cotton flakes", but both represent the enlargement of sebaceous glands. Furthermore, we propose that the rainbow-like image observed on dermoscopy corresponds to the basaloid cell component. To the best of our knowledge, this is the first time the term "cotton flakes" has been used in the dermoscopy description of a sebaceous adenoma, and our report represents the first instance of a rainbow-like image observed on dermoscopy for this neoplasm. While clinical and dermoscopic features may give rise to suspicion of sebaceous adenoma/sebaceoma, a definitive histopathologic diagnosis is crucial for accurate identification [7].

On a histological level, sebaceous adenoma tumor lobules exhibit varying layers of germinative, immature sebocytes at the periphery. These sebocytes show mitotic activity but lack significant cell atypia. Sebaceous adenomas also demonstrate variably expanded basaloid cells, exceeding the typical two-cell layers observed in sebaceous glands and sebaceous hyperplasia. The differential diagnosis of sebaceous adenoma involves distinguishing it from clear cell variants of eccrine, melanocytic, keratinocytic, or xanthomatous lesions, as well as renal cell carcinoma [5,7-9].

Typically located in the superficial dermis, sebaceous adenoma can raise suspicion for well-differentiated sebaceous carcinoma if it extends into the deep dermis, exhibits high mitotic activity, and mild cytologic atypia [5]. Differentiating well-differentiated sebaceous carcinoma from sebaceous adenoma can be challenging in rare cases. When in doubt, complete excision of any sebaceous tumor is recommended. The cystic variant of sebaceous adenoma serves as a marker for MTS and should be stained for mismatch-repair (MMR) protein deficiency proteins and immunohistochemical markers (MSH2, mismatch repair gene). The diagnosis of the syndrome is based on the presence of at least one sebaceous neoplasm and a visceral malignancy or, alternatively, multiple keratoacanthomas associated with visceral malignancies as colorectal, genitourinary, and breast carcinomas and a family history of MTS [7,8,10].

Traditionally, sebaceous adenomas are not linked to significant local recurrence, aggressive behavior, or metastasis. Most practitioners prefer a conservative but complete excision of these lesions, which appears to be an appropriate therapeutic approach [10].

## Conclusions

This case emphasizes the significance of dermoscopy in aiding clinical diagnosis and underscores the association of these tumors with MTS. Early identification and appropriate management, including complete excision, play a crucial role in ensuring favorable outcomes.
